# Attempting to distinguish between endogenous and contaminating cytokeratins in a corneal proteomic study

**DOI:** 10.1186/1471-2415-11-3

**Published:** 2011-01-27

**Authors:** Mikkel Lyngholm, Henrik Vorum, Kim Nielsen, Niels Ehlers, Bent Honoré

**Affiliations:** 1 Department of Ophthalmology, Aarhus University Hospital, Nørrebrogade 44, 8000 Aarhus C, Denmark; 2 Department of Medical Biochemistry, Aarhus University, Ole Worms Allé 3, Bldg. 1170, 8000 Aarhus C, Denmark; 3 Department of Ophthalmology, Aalborg Hospital, Aarhus University Hospital, Hobrovej 18-22, 9100 Aalborg, Denmark

## Abstract

**Background:**

The observation of cytokeratins (CK's) in mass spectrometry based studies raises the question of whether the identified CK is a true endogenous protein from the sample or simply represents a contaminant. This issue is especially important in proteomic studies of the corneal epithelium where several CK's have previously been reported to mark the stages of differentiation from corneal epithelial stem cell to the differentiated cell.

**Methods:**

Here we describe a method to distinguish very likely endogenous from uncertain endogenous CK's in a mass spectrometry based proteomic study. In this study the CK identifications from 102 human corneal samples were compared with the number of human CK identifications found in 102 murine thymic lymphoma samples.

**Results:**

It was anticipated that the CK's that were identified with a frequency of <5%, *i.e. *in less than one spot for every 20 spots analysed, are very likely to be endogenous and thereby represent a 'biologically significant' identification. CK's observed with a frequency >5% are uncertain endogenous since they may represent true endogenous CK's but the probability of contamination is high and therefore needs careful consideration. This was confirmed by comparison with a study of mouse samples where all identified human CK's are contaminants.

**Conclusions:**

CK's 3, 4, 7, 8, 11, 12, 13, 15, 17, 18, 19, 20 and 23 are very likely to be endogenous proteins if identified in a corneal study, whilst CK's 1, 2e, 5, 6A, 9, 10, 14 and 16 may be endogenous although some are likely to be contaminants in a proteomic study. Further immunohistochemical analysis and a search of the current literature largely supported the distinction.

## Background

Cytokeratins (CK's) belong to the family of intermediate filaments, and are expressed in a variety of different cell types, including those of the eye. CK's can be classified into type I (acidic) and type II (basic), and they often appear together as pairs of these two types of proteins [1,2]. Within the eye in particular, CK's have been shown to be important proteins with regard to cellular development, proliferation and differentiation [3-7].

Proteomics using two-dimensional gel electrophoresis (2D-PAGE) in combination with mass spectrometry (MS) is often used as a screening tool in the search for differentially expressed proteins [8]. If CK's are the subject of such an experiment, an important issue to determine is whether the CK originates from the investigated tissue or whether its presence is a consequence of contamination from the laboratory environment.

As a consequence of the very high cell turn-over of the surface epithelia the environment is contaminated with cells from hair, skin, nails, eyebrows, eyelashes, airways, etc. Furthermore, CK's remain ubiquitous contaminants even in laboratories with very high cleaning standards being extremely difficult to eliminate [9], though their presence in MS laboratories should be reduced as much as possible [10]. One way to diminish the environmental contribution of CK's is to filter all the liquids used for 2D-PAGE, including the reducing agents [11] and to have efficient laboratory protocols to maintain clean conditions in general [12].

In the search for stem cell markers, we previously investigated the differences in protein expression between the central corneal epithelium and the limbal epithelium by a proteomic approach, in an effort to identify proteins either highly expressed or exclusively present in limbal epithelium [13]. The limbus, which is located between the conjunctiva and the cornea, is a niche for corneal epithelial stem cells. Several CK's have been proposed as markers of stem cells, transient amplifying cells or differentiated cells [5,7,14]. Therefore, we wanted to include biologically significant CKs in the investigation in the search for limbal stem cell markers.

This study attempts to discuss whether it is possible by a simple procedure to distinguish between very likely endogenous and uncertain endogenous CK's by counting and evaluating all the human CK's identified by mass spectrometry in a human and murine study. These results are compared with previous reports on location and distribution of CK's in the ocular surface tissue. Further examination was performed by evaluating the specific mass spectra of the cytokeratins in conjunction with immunohistochemistry.

## Methods

### Human sample collection and preparation

Seven human eyes were obtained from The Institute of Anatomy, Aarhus University within 48 hours post mortem. The study adhered with the guidelines from the local ethical committee and the tenets of the Declaration of Helsinki. The eyes were carefully rinsed in sterile isotonic saline and the epithelium was marked with 8 and 10 mm trephines prior to loosening the epithelium by dabbing with 70% alcohol. Under clean conditions, the corneal epithelium was gently scraped to separate the central 8 mm epithelium. The intermediate epithelium (8-10 mm ring) was discarded and the limbal fractions were considered to be the epithelium scraped outside the 10 mm ring since, the conjunctival epithelium was not able to be as easily loosend by this method. Both fractions were transfered to lysis buffer, pH 3-10 NL [13].

### Mouse sample collection and preparation

Two spontaneously developed thymic lymphomas, SM5 and SM7, from C57BL/6J-Trp53tm1Tyj mice deficient for the p53 gene were explanted, *in vitro *cultured, and established as cell lines growing in RPMI-1640 culture medium supplemented with 10% fetal calf serum and 50 μM 2-mercaptoethanol. Freshly prepared thymocytes obtained from pools of five mice (either C57BL/6J-Trp53tm1Tyj or normal C57BL/6) and cultured SM5 and SM7 cells were washed extensively in PBS and subsequently freeze dried. The cell pellets were dissolved in lysis buffer, pH 3-10 NL [8].

### 2D-PAGE and MS identification

2D-PAGE and silver staining was performed as previously described [8,13]. The gels were re-hydrated and the cellophane sheets peeled off prior to protein gel spot excision. Gel pieces were dehydrated in acetonitrile, dried and the proteins reduced for 1 h at 56°C in 10 mM dithiotreitol (DTT) and 100 mM NH_4_HCO_3_. The solution was exchanged with 55 mM iodoacetamide in 100 mM NH_4_HCO_3 _for 45 min. Gel pieces were then washed in 100 mM NH_4_HCO_3_, dehydrated in acetonitrile, rehydrated in 100 mM NH_4_HCO_3_, dehydrated in acetonitrile, dried and swelled in digestion buffer (50 mM NH_4_HCO_3_, 5 mM CaCl_2 _and 12.5 ng/μl trypsin Gold (mass spectrometry grade; Promega, Madison, WI, USA). The digestion was performed overnight at 37°C prior to peptide extraction by 1 change of 20 mM NH_4_HCO_3 _and 3 changes of 5% formic acid in 50% acetonitrile. The sample was dried and peptides resuspended in buffer A (water/acetonitrile/formic acid, 97.7/2/0.3, V/V/V). The peptides were separated on an inert nano LC system of a Famos micro autosampler, a Switchos micro column switching module and an Ultimate micro pump from LC Packings (San Francisco, CA) before MS analysis. Samples were concentrated and desalted on a 300 μm inner diameter x 5 mm precolumn (LC Packings) packed with 5 μm C18 PepMap100 material. A 75 μm inner diameter x 15 cm Nano column packed with 3 μm C18 PepMap100 material was used to separate the peptides. Gradient elution from the column was performed by mixing decreasing volumes of buffer A with increasing volumes of buffer B (water/acetonitrile/formic acid, 9.7/90/0.3, V/V/V). The peptides were eluted into the nano electrospray ion source of the quadrupole time-of-flight mass spectrometer (Micromass, Manchester, UK). MS survey scans were acquired using MassLynx 4 SP4 (Waters). The instrument was operated in a data-dependent MS to MS/MS switching mode. Doubly, triply and quadruply charged peptide ions detected in MS survey scans triggered a switch to MS/MS for obtaining peptide fragmentation spectra. The processed data were used to search in the Swiss-Prot Database (version 56.9) using the on-line version of the Mascot MS/MS Ion Search facility (Matrix Science, Ltd., http://www.matrixscience.com) [15]. Searching was performed with doubly and triply charged ions with 2 missed cleavages, a peptide mass tolerance of 50 ppm, one variable modification, Carbamidomethyl-C and an MS/MS tolerance of ± 0.02 Da. Only human proteins identified by bold red peptides were regarded as significant and reported (excluding duplicate homologous proteins).

### Evaluation of the LC-MS/MS identifications

All significant human CK hits from the Mascot search were counted in each sample of the human and mouse study (Table 1) and the mass spectra were evaluated. The CK's were grouped as very likely endogenous (observed in less than 5% of the spots) or uncertain endogenous (observed in more than 5% of the spots) on the basis of the observed frequency in the human study.

**Table 1:** Frequency of human cytokeratins identified in spots from 2D-PAGE in a human corneal study and mouse thymic lymphoma study.

CKs	Type I(acidic)/II(neutral or basic)	Previously described in human non-tumour-tissue	Previously described in ocular epithelium	**Location**^**1**^	Total count in 102 mouse spots - %
*Very likely endogenous (observed in <5% of human samples)*					
3	II		Cornea^19,17^	B	0%
4	II	Sebaceous glands, airway epithelium^19^	Conjunctiva^17^, cornea^5^	B	4%
7	II	Glands^19^	Central basal cornea^17^No found^20^	-	0%
8	II	Glands^19^	Cornea^21^, conjunctiva^17^	-	0%
11	I	Epidermis^19^		-	0%
12	I		Cornea^19,2^	C	0%
13	I	Glands, airway epithelium^19,2^	Conjuntiva^25^, cornea^5^	B	2%
15	I	Basal keratinocytes^2^	Limbal basal^7^	L	2%
17	I	Hair follicle, nails^2^	Cornea^17^	-	0%
18	I	Glands, simple epithelium^19,2^	Cornea^21^	-	0%
19	I	Glands, airway epithelium^19^	Conjunctiva^4^, basal limbus^2^	L	0%
20	I	Gastrointestinal tract epithelium^2^		-	0%
23	I	Non (found in pancreatic tumours)^22^		-	0%
*Uncertain endogenous (observed in >5% of human samples)*					
1	II	Epidermis^19^		B	36%
2e	II	Epidermis^19^		B	78%
5	II	Epidermis, glands, airway epithelium, hair follicles^19^	Conjuntiva^25^, cornea^19, 24^	B	3%
6A	II	Glands, airway epithelium, hair follicles^19^		B	27%
9	I	Palmoplantar epidermis^2^		B	99%
10	I	Epidermis^19^		B	75%
14	I	Epidermis, glands, airway epithelium, hair follicles^19^	Conjuntiva^25^, cornea^17^	B	18%
16	I	Epidermis^19^	Cornea^4^	B	6%

^1^Location: C, central fraction, L, limbal fraction, B, both limbal and central fraction

### Immunohistochemistry

Antibodies against CK 3/12 and 19 were obtained from Chemicon (Billerica, MA), and antibodies for CK 15 were obtained from Santa Cruz Biotechnology (Santa Cruz, CA). The immunohistochemical protocol has previously been published [13].

### Statistical analyses

An unpaired non-parametric test (Mann-Whitney) was performed to test for significant differences between CK expression in the human and mouse studies.

## Results and Discussion

**Figure 1:**
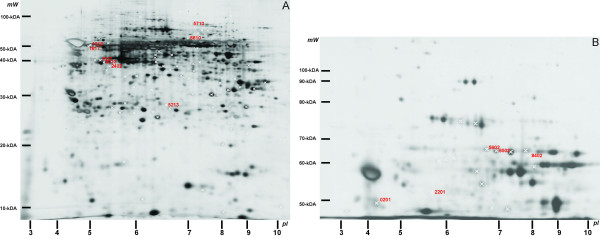
**2D-gels (A:12%; B:6%) from the limbal fraction shows the significant spots (marked with the SSP number)**. The approximate pI and molecular mass is indicated on the axes. SSP No. 0103 and 1205 were not expressed on this gel (B:6%).

We hypothesised that the higher the frequency that a certain CK is identified by MS from a group of 2D-PAGE spots the more likely it is to be a contaminating protein. A set of 102 spots from the human study [13] (Figure 1) was included in the investigation in conjunction with a set of 102 mouse spots [16]. In Table 1, CKs are listed in two groups based on the observed frequency of the CK in the human study; very likely endogenous (observed in less than 5% of the spots) and uncertain endogenous (observed in more than 5% of the spots) together with previously published information on human tissue expression of each CK. The corresponding frequencies in the mouse study are also shown. Thirteen CK's were either not detected at all in any spot or found in less than 5% of the spots in the human study; CK's 3, 4, 7, 8, 11, 12, 13, 15, 17, 18, 19, 20 and 23 correlating with the mouse study (<5%). Eight CK's were identified in more than 5% of the human samples; CK's 1, 2e, 5, 6A, 9, 10, 14 and 16. These were also seen often in the mouse study except for CK5, which was observed with a frequency of 3%. There were no differences between the CK expression in the human and mouse study in the combined groups (p = 0.51), the group of very likely endogenous (p = 0.34) or the group of uncertain endogenous (p = 0.72). We expected to find some of the very likely endogenous CK's in the mouse study since several of these proteins, in addition to the expression in the human corneal epithelium, also are found in skin, hair and other tissues, largely explaining the source of contamination from the laboratory environment. Table 2 shows the theoretical and observed molecular mass (M) and pI of each very likely endogenous CK that was identified in the human study. In general, a higher observed molecular mass than theoretical molecular mass may indicate post-translational modification, the protein is a precursor or alternatively a contaminant. A lower observed molecular mass than theoretical molecular mass is likely to be a cleavage product. The observed molecular masses listed in Table 2 are largely equal to or below the theoretical masses. The coverage of each observed CK in the likely endogenous group is shown in Table 2 together with the coverage from the first amino acid in the most N-terminal identified peptide to the last amino acid in the most C-terminal identified peptide in the protein. In none of the cases do we find the fraction of protein covered to be significantly larger than in accordance with the observed molecular mass, in keeping with the identifications being very likely endogenous. Identifications tend to be more reliable when the sequence coverage is large or based on a few high quality spectra. Only identifications "in bold red" were included (see above).

**Table 2:** Characterization of some very likely endogenous cytokeratins.

CKs	Spot number in gels (6%=',12%=*)	UniProtKB/Swiss-Prot	Observed M (kDA)	Theoretical M (kDA)	Observed pI	Theoretical pI	Coverage (%)	**Fraction of protein covered (%)**^**1**^
3	6503'	K2C3_HUMAN	64	64.5	6.90	6.12	12	56
	5213*	P12035	28		6.60		3	34
	5610*		60		7.00		31	88
	5713*		80		7.05		9	56

4	8402'	K2C4_HUMAN	62	57.3	7.80	6.25	6	21
	5602'	P19013	65		6.70		4	57
	5610*		60		7.00		3	24

12	0201'	K1C12_HUMAN Q99456	51	53,4	4.30	4.70	9	40

13	0301'	K1C13_HUMAN	56	49.6	4.40	4.91	25	71
	1205'	P13646	56		4.80		35	71
	1617*		50		5.05		16	64
	1618*		50		5.10		34	90
	2402*		38		5.50		5	19

15	2201'	K1C15_HUMAN P19012	52	49.2	5.60	4.71	16	78

19	2513*	K1C19_HUMAN	39	44.1	5.40	5.04	23	81
	1517*	P08727	40		5.30		17	81

^1^This was determined by calculating the fraction of the protein covered from the first amino acid in the most N-terminal peptide to the last amino acid in the most C-terminal peptide observed.

### Very likely endogenous CK's

**Figure 2:**
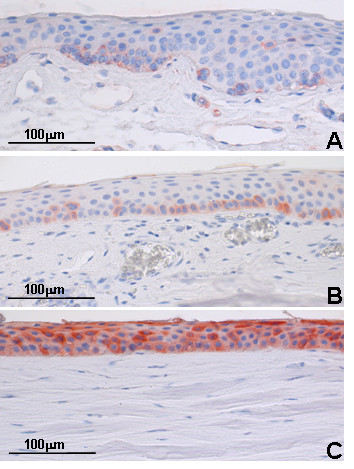
**Immunohistochemical staining against various antigens**. (**A**) CK 15 and (**B**) CK 19 appear in the limbal epithelium. (**C**) CK 3/CK 12 stainings in central corneal epithelium.

**Figure 3:**
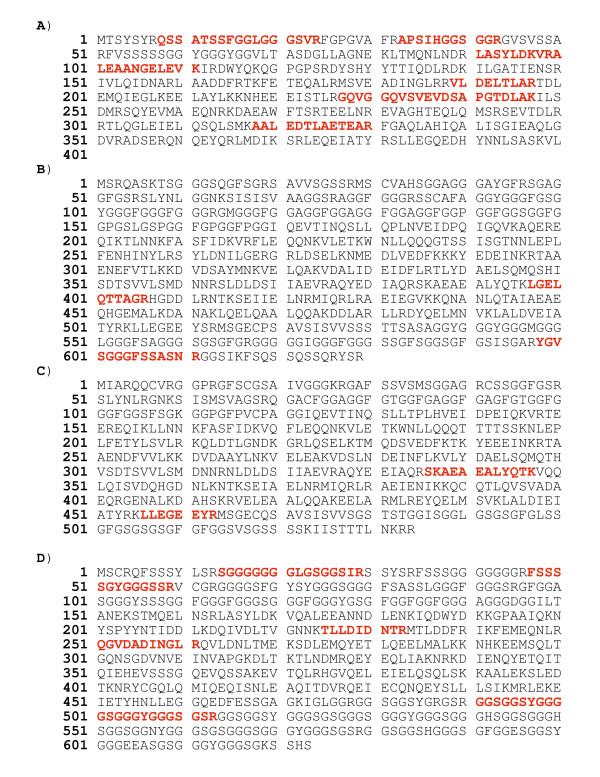
**Peptide sequences from the Mascot search results**. **A**: CK 19 identified in a 39-kDa spot by 7 peptides (Matched peptides shown in bold red). It is most likely an endogenous protein although a contaminating protein cannot be excluded. **B**: Two peptides (24 amino acids) identify CK 3 from a spot focused around 28 kDa. This could be an endogenous protein or it could be due to contamination of the samples. **C**: Two peptides (20 amino acids) identify CK 4 from a spot focused around 60 kDa. This could be an endogenous protein or it could be a contamination of the samples. **D**: CK 9 identified by the matched peptides throughout the whole protein. Since the sample originates from a spot focused around 10 kDa in the gel the protein is regarded as a contaminant.

Among the CK's observed in less than 5% of the human samples, CK 15 (49 kDa) was identified in one spot from the limbal epithelial fraction in the 50-kDa area. The proteomic identification was confirmed by immunohistochemical staining (Figure 2A). Indeed, CK 15 has recently been found in the basal limbal epithelium [7]. CK 19 (44 kDa) was identified in 2% of the spots, all focused in the 40-kDa area in the limbal epithelial fractions (Figure 1). CK 19 is a well-characterized marker for basal limbal epithelial cells (Figure 2B), and is furthermore found in simple epithelia and epidermal appendages [2,5,17]. CK 15 and CK 19 were regarded as very likely endogenous because of the distinct expression in only the limbal fraction, and also because they were identified in only a few spots (<2%). In addition, both proteins were identified by peptides covering around 80% of the protein as defined by the most N-terminal to the most C-terminal peptides observed (Table 2, Figure 3A). Human CK 15 was also observed in two mouse samples. CK 15 is known to be expressed in basal epithelia and hair follicle cells [18], which thus are likely to be rare contaminants in the laboratory environment.

CK 3 and CK 12 were identified in 4% and 1% of the human samples respectively and are both specific markers for differentiated corneal epithelial cells (Figure 2C) [5,17,19]. A mass spectrum from a 28-kDa spot identified CK3 by two peptides from the C-terminal of the protein (64.5 kDa) and thereby could be an identification of an endogenous protein. However, we cannot exclude that this is a cleaved contaminating protein introduced before electrophoresis (Figure 3B).

CK 4 and CK 13 were identified in 3% and 5% of the spots in the human set. Both proteins have previously been identified in the ocular surface (immunohistochemically), in addition to dermal glands and airway epithelium [2,5,19]. A 60-kDa spot was identified as CK 4 by two peptides in the C-terminal part of the protein (Figure 3C). This could be a cleaved endogenous protein, however, contamination cannot be excluded. CK 4 and CK13 were observed in 4 and 2 samples in the mouse study respectively, indicating that they may be rare contaminants.

Some of the keratins were rarely identified, some were not observed and among these some have to our knowledge not been reported to be localized in the cornea, i.e. CKs 11, 20 and 23 (table 1). Thus, if identified, they would be very likely endogenous CK's[20-22].

### Uncertain endogenous CK's

CK 16 appeared in 6% of the spots in both the human and the mouse study. It is expressed in palmoplantar epidermis, in epidermal appendage and in mucosa [2,19,23]. However, CK 16 has also been shown to be expressed in corneal epithelium to a minor extent [4]. None of the mass spectra proved useful in determining the origin of the contamination (data not shown).

CK 5 and CK 14 are paired CK's and were present in 12% and 19% of the samples respectively. CK 5 and CK 14 have previously been found in the corneal epithelium [17,24], however, they are also present in basal keratinocytes in epidermis [2,19,23]. Therefore, most of the identified CK 5 and CK 14 are considered as contaminants, though a few of the identifications could be endogenous.

CK 6A was identified in 22% of the spots and was most likely a contaminant. CK 6A is expressed in palmoplantar epidermis, epidermal appendage and mucosa [2,19,23]. Again, none of the mass spectra aided in determining the origin of the contamination (data not shown).

CK's 1, 2e, 9 and 10 were identified in more than 73% of the human spots. Figure 3D shows the peptide-sequence leading to identification of CK 9 in a spot focused at around 10 kDa in the gel. Fragments of peptides were identified throughout the whole protein (62 kDa). It entails that the identification was based not only on a 10-kDa fragment, but rather on the whole protein. This can only be explained by contamination with an un-cleaved protein located on the surface of the excised spot or contamination introduced into the sample prior to enzymatic digestion. All 4 keratins are abundantly expressed in suprabasal cells of stratified and cornified epithelia or palmoplantar epidermis [2,19,23], but not in the corneal epithelium [4,17,25]. CK's 1, 2e, 9 and 10 are all considered to be contaminating. This is consistent with previous reports [26,27].

### Aspects of contamination

Contamination of CK's occur principally during sample collection or gel preparation and excision. Indeed, by our scraping method there is a risk of contaminating the limbal epithelial fraction by conjunctival epithelium. Immunohistochemistry is especially effective in revealing this type of contamination[13]. If contamination occurs during sample collection and preparation prior to loading on the gels, the contaminating CK's may be focused as spots in the gels. If the contamination occurs during the preparation of the gel solutions, gel casting, spot excision or in the preparation of the spots prior to digestion, the contaminants may appear as identified proteins with molecular masses not in accordance with the spot position.

### Impact of the number of investigated spots

The total number of investigated spots is important in this set-up. The more frequent a CK is identified the more likely it is to be a contaminant. However, it should also be considered that endogenous CK's that have several isoforms or have undergone degradation could erroneously be interpreted as contamination and be excluded from the investigation.

### Optimization of the method

In a proteomic screening experiment, it may be desirable to obtain as many protein candidates as possible if specific validation techniques are to be subsequently undertaken. An important issue is how much of the sample should be loaded onto the gel. Overloaded gels may lead to increased spot size that can merge with neighbouring spots making further analysis potentially difficult and inaccurate. Conversely, if an inadequate amount of sample is loaded onto the gel, the relative amount of contaminating proteins will tend to increase. In the present study, a relatively small amount of protein was loaded, in an effort to preserve the resolution of proteins in the 40-60 kDa range where gel spots have a tendency to merge together. However, despite the gels produced well-focused spots with low background staining the number of successful identifications was lower than expected, probably as a result of the relatively small amount of protein loaded and the relatively high frequency of contaminating CK's.

## Conclusions

It is very important to consider possible contamination of CK's when undertaking MS based proteomic screening experiments, especially with regard to studies of the cornea. Using proteomics as a screening tool in the search for differences in protein expression, including CK's, careful consideration should be applied to the sample amount chosen. Generally one should load as much sample as possible, to the point of not compromising 2D-gel spot resolution. We have shown that if several 2D-gel spots are being processed, CK's represented in less than 5% percent of the spots are very likely to be endogenous identifications (CK's 3, 4, 7, 8, 11, 12, 13, 15, 17, 18, 19, 20 and 23). If a CK is identified in a large part of the samples it is an uncertain endogenous CK (CK's 1, 2e, 5, 6A, 9, 10, 14 and 16). Confirmation of the CK identification by other biochemical methods such as immunohistochemistry is also very important.

## Competing interests

The authors declare that they have no competing interests.

## Authors' contributions

ML carried out the 2D PAGE analysis and the immunohistochemistry and contributed substantially to the writing. HV carried out the 2D PAGE and contributed to the writing. KN contributed substantially to the immunohistochemistry and the writing. NE participated in its design and coordination and helped to draft the manuscript. BH carried out the mass spectrometry and contributed substantially to the writing. All authors read and approved the final manuscript.

## Pre-publication history

The pre-publication history for this paper can be accessed here:

http://www.biomedcentral.com/1471-2415/11/3/prepub
